# Supplementary material for: Training forecast to football athletes using Hopfield neural networks based on Markov matrix
Source: PLoS One. 2025 Jun 12;20(6):e0317604. doi: 10.1371/journal.pone.0317604 (PMC12161553; doi:10.1371/journal.pone.0317604)
Supplement: Attachment — Submitted filename: R2-responseLetter.docx [file pone.0317604.s004.docx]

**Dear Editor and Reviewers,**

Many thanks for your insightful and informative comments. We have revised this manuscript according to the reviewers’ comments and gave the detailed responses to their questions one by one in the following “Response to Reviewers’ comments”.

Best regards,

Yours sincerely,

Hongxing Peng

**Reviewer #1: This paper proposes a neural network based on the Markov probability transition matrix to predict the training performance of football athletes. This paper has been revised according to the previous comments. However, the readability and writing are unsatisfactory.
Some comments are as follows:
1. The language should be polished. The logic in Section 1 Introduction is confused.**
Many thanks for the insightful comments. The revision was marked with the red.

**Response:**

We rewrote the Section 1 Introduction and organized the section to understand the logical. Please see Line #30 to Line #92.

**2. The quality of Figure 1 “The structure of DHNNs.” is unsatisfactory.**

**Response:**

We re-drew the Fig. 1, please see Line #199.

**3. The control scheme seem to be simple validate of the existing predictive algorithm. The highlights of the work are unobvious.**

**Response:**

We supplemented two evaluated metrics Accuracy and G-score, then we compared our model with the four competitors. Additionally, we conducted statistical analysis on these results. Please see Line #326 to Line #340.

**4. The simulation process is too simple. Only bar charts are provided cannot be accepted.**

**Response:**

We supplemented the experiments in Section 5.1. Our goal is to analyze the factors that affect athlete training performance of football athletes, therefore, we used the SPSS tool to analyze the relations between the seven training indicators and the forecast training performance. Please see Line #293 to Line #298.

To further observe the seven training indicators, we used k-means clustering to cluster the seven training indicators. Please see Line #308 to Line #314. Meanwhile, we visualized the clustered results in Fig. 3 in Line #319.

Additionally, we analyzed the training loss and testing loss of our model in Line #315 to Line #318. And please see the Fig. 4 in Line #324.
