# Supplementary material for: Training forecast to football athletes using Hopfield neural networks based on Markov matrix
Source: PLoS One. 2025 Jun 12;20(6):e0317604. doi: 10.1371/journal.pone.0317604 (PMC12161553; doi:10.1371/journal.pone.0317604)
Supplement: Attachment — Submitted filename: responseletter-2024-9-30.docx [file pone.0317604.s003.docx]

**Dear Editor and Reviewers,**

Many thanks for your insightful and informative comments which are very helpful for the further improvement of this work. We have revised this manuscript according to the reviewers’ comments and gave the detailed responses to their questions one by one in the following “Response to Reviewers’ comments”.

Best regards,

Hongxing Peng

**Editor Comments:**

**1. Add more new contributions to the manuscript**

**Response:**

Many thanks for the insight comments. The revision was marked with the red.

New contributions are added, and please see Line #78 to Line #81.

**2. Apply Evaluation metric and testing strategies to the proposed model**

**Response:**

We applied two evaluation metrics Precision and F1-score to test the forecast ability of the proposed model, please see Section 5.2 in Line # 295 to Line #304. Additionally, we compared the running efficiency of the proposed model with that of the competitors. Please see Section 5.3 in Line #305 to Line #318.

**3. Technically paper is sound.**

**Response:**

Many thanks for the comments.

**4. Use technical writing for improving the quality of manuscript.**

**Response:**

We revised carefully this paper and made some minor adjustments to highlight the contributions.

**5. The manuscript will be accepted after making changes.**

**Response:**

Many thanks for the comments
